# All That Glitters, Etc.

**Published:** 1892-10

**Authors:** 


### ALL THAT GLITTERS, ETC.

In our last issue in speaking of the possibilities of cholera invasion, notwithstanding the rigid quarantine at New York and elsewhere, it was said that sometimes ignorance on the part of a guard, sometimes a bribe, would occasionally account for the appearance of a disease inside the lines. It has also been said that as the strength of a chain is the strength of its weakest link, so the strength of a quarantine may be estimated by the strength of its weakest part. There are ways and ways of evading quarantine; and there are ways and ways of disseminating infection. One method not laid down in the list was illustrated here about during the small pox period last winter. The story is told by our own health officer who vouches for its truth.

After the last case was convalescent (or dead, the doctor forgot to say which), the old colored man who had been acting as nurse, was told to take out all the infected bedding and cots, etc., and burn them. He did so promptly,(?) and nothing more was heard of small pox; no new cases occurred and camp was broken up; the disease had been "stamped out" they said, and the subject was forgotten.

Several weeks later old Uncle Dick, the colored man, sent for the doctor, very sick. Much to the doctor's surprise, he found it to be a case of varioloid in full bloom. Here was a mystery. Where had he been? "No where Marster, just staid right at home ever since I quit nussin' de small pox." The doctor was puzzled, but he knew there was a connection somewhere, and recently; so he inquired "Dick, did you burn up all those blankets and quilts and pillows and things as I told you?" Dick looked guilty, and after awhile, stuttered out, "Marster, I knows youse gwine to kill me, and maybe I deserves it, but one of dem new blankets looked so clean and white, and de cold wedder was coming on, so I jist 'zempted dat one from de fire, and I

been kiverin' up with it; I specs dat how I come by de sickness. Marster is I gwine die you reckon?" "I hope so," said the doctor, "you ought to," (but Dick's alive and tells the story as a good joke on the doctor; fact).

